
JOURNAL INFORMATION
==============================
NLM Title Abbreviation: Atlanta Med Surg J (1884)
NLM Journal ID: 101251623

Atlanta Medical and Surgical Journal (1884)

Publisher: Biomedical Journal Digitization Project

ARTICLE INFORMATION
==============================
PMCID: PMC9201869
PMID: 35829614
Article version: 1
Subjects: Original Communications

What Kind of Shoes We Should Wear* A talk delivered before the Atlanta Society of Medicine, January 19, 1899.

Robinson G. P. M.D. Atlanta, Ga.

Print publication date: 1899 Feb
Volume: 15
Volume Series: New Ser
Issue: 12
First page: 810
Last page: 812
License: This work is free of known copyright restrictions. For more information, please see PMC Back Issue Digitization.
License URL: https://creativecommons.org/publicdomain/mark/1.0/

==============================

observed through green. If only the left eye is doing the work, only the green letters will be seen. I prefer the latter test, as no errors can arise in making it. This test is made at a distance of fifteen or twenty feet. Place the colored letters in front of a window, candle or gas-jet.

I have succeeded in establishing binocular vision in only a few cases where the muscle had been set back by tenotomy. It is apropos to state that, owing to the tediousness of these cases, and the time and patience required, very few cases of this character are undertaken. These patients must go through life possessing two good eyes, normal in acuteness, and using only one.

The only way to avoid this very undesirable condition of affairs is to steer clear of tenotomies, and rely upon the operation of shortening the so-called weak muscle.

In a future article I propose to take up the operation of shortening a muscle, and the methods used in restoring binocular vision.

*507 English-American Building.*

---

### WHAT KIND OF SHOES WE SHOULD WEAR.*

BY G. P. ROBINSON, M.D.,

ATLANTA, GA.

I want to say just a few words in regard to the abuse that exists in regard to the covering of our children's feet, and am anxious to gain your cooperation and assistance in doing away with the present narrow and so-called fashionable shoe. I hope you will call the attention of parents to the bad shape of the ordinary shoe now worn, and the possibilities of deformity and pain in the foot as the little ones reach maturity.

Dr. Dean has shown that the infant, instead of being flat-footed, as is ordinarily taught, has, on the contrary, the arch well formed. The anatomy of the foot allows it at this age to easily bend up against the tibia on account of the laxity of the tendo achillis,

*A talk delivered before the Atlanta Society of Medicine, January 19, 1899.

and the stretching of the plantar fascia gives it a strong resemblance to flat-foot. As the child begins to walk, the internal arch begins to break down, which lasts until the third year. The arches of both feet break equally. When they do not break down they are presumably of light weight. From the third year on the arch is rebuilt, one foot improving before the other, the female building before the male; at the eighth year the adult type is complete.

These facts should all be borne in mind, especially the ease with which the arch is broken down, and that it is a physiological fact. A child should not be forced to walk prematurely; they will try their feet and walk sufficiently of their own accord for physiological development. Training children to turn out their toes puts the arch in such position that the muscles give it the least support.

The phalanges of the great toe do not point naturally toward the outer border of the foot; common as this condition is in the adult, it begins in the child's first shoes. Infant's and children's shoes should be rights and lefts, and should be broad enough at the toes not to compress either the great or little toe. The shoes should be comfortable and leave sufficient room for development, and not crowd the great toe toward the median line or interfere with the proper elasticity of the foot. The elasticity of our step depends on the firm pressure on the great toe and the raising of the body on it as support. Hence, any interference in the normal position of the great toe may be seen in the walk.

The ordinary child's shoe on the market is symmetrical and equidistant from the median line. This shape crowds either the great toe toward the outer line of the foot or the little toe toward the median line, or it crowds the great toe outward and upward, causing that distressing condition known as hammer-toe. I have made some tracings of feet of children who have run bare-footed much of their life. You will notice how straight the great toe is. The inside line of the foot is nearly straight, widening inward slightly at the great toe. If you draw a straight line through the heel and instep and then carry that line straight through the plantar surface of the foot, you will find that line runs nearly parallel with the inside line of the foot. Notice, also, the apparent slight curve in the whole foot. In quite young children you will notice the great breadth at the toes. This fact should be borne in mind in buying shoes for very young children. Be sure to get ample width at the toes, the inside line of shoe should be straight. The ordinary infant's shoe sold has no right or left, nor are the lines right. A line drawn through the center of the heel and shank (instep) of the shoe and continued on through the shoe will be found to be equidistant and symmetrical throughout. This is very bad for the young growing foot. This same fault is also seen in the pointed shoe of older children. I have talked with Mr. Carlton and he has put in a line of shoes of much better shape. I sincerely hope the profession will advance this missionary work as far as lies in their power, and let our little children grow to adult life with their feet in their natural and normal shape, and not pinched and crowded into misshapen shoes, causing torture but little short of such as Chinese children endure.

*308 English-American Building.*

---

THE *Centralblatt für Chirurgie* publishes an article by S. Kofmann describing a method of producing local anesthesia by producing a local anemia by the application of an elastic tourniquet. He reports several cases in which the results were quite satisfactory. Foreign bodies were removed from the hand. Ingrowing nails from the toes. Tumors removed, abscesses opened, etc. He claims to be so pleased with the method that he now rarely operates on the extremities in any other way. The extremity is raised, the elastic ligature applied and the operative area cleaned. The constriction should be complete and the anemia marked, the skin white and bloodless. The cases reported mention complaints of pain due to the constriction of the elastic ligature, which the writer seems to consider as unimportant. The operation must not be begun at once after the application of the tourniquet, but time must be allowed for the anemia and anesthesia to appear.

---

***NOTICE.—This JOURNAL and THE INTERNATIONAL JOURNAL OF SURGERY one year for $1.00 cash.***
